# Inflammatory-Induced Hibernation in the Fetus: Priming of Fetal Sheep Metabolism Correlates with Developmental Brain Injury

**DOI:** 10.1371/journal.pone.0029503

**Published:** 2011-12-29

**Authors:** Matthias Keller, David P. Enot, Mark P. Hodson, Emeka I. Igwe, Hans-Peter Deigner, Justin Dean, Hayde Bolouri, Henrik Hagberg, Carina Mallard

**Affiliations:** 1 Neonatology, Department of Pediatrics I, Essen University Hospital, Essen, Germany; 2 Australian Institute for Bioengineering and Nanotechnology, University of Queensland, St Lucia, Australia; 3 Biocrates Life Sciences AG, Innsbruck, Austria; 4 Fraunhofer IZI EXIM Rostock and Department of Nephrology, Rostock University, Rostock, Germany; 5 Department of Neuroscience and Physiology, Sahlgrenska Academy, University of Gothenburg, Gothenburg, Sweden; 6 Institute of Reproductive and Developmental Biology, Department of Surgery and Cancer, Imperial College, London, United Kingdom; Institute of Clinical Medicine, National Cheng Kung University, Taiwan

## Abstract

Prenatal inflammation is considered an important factor contributing to preterm birth and neonatal mortality and morbidity. The impact of prenatal inflammation on fetal bioenergetic status and the correlation of specific metabolites to inflammatory-induced developmental brain injury are unknown. We used a global metabolomics approach to examine plasma metabolites differentially regulated by intrauterine inflammation. Preterm-equivalent sheep fetuses were randomized to i.v. bolus infusion of either saline-vehicle or LPS. Blood samples were collected at baseline 2 h, 6 h and daily up to 10 days for metabolite quantification. Animals were killed at 10 days after LPS injection, and brain injury was assessed by histopathology. We detected both acute and delayed effects of LPS on fetal metabolism, with a long-term down-regulation of fetal energy metabolism. Within the first 3 days after LPS, 121 metabolites were up-regulated or down-regulated. A transient phase (4–6 days), in which metabolite levels recovered to baseline, was followed by a second phase marked by an opposing down-regulation of energy metabolites, increased pO_2_ and increased markers of inflammation and ADMA. The characteristics of the metabolite response to LPS in these two phases, defined as 2 h to 2 days and at 6–9 days, respectively, were strongly correlated with white and grey matter volumes at 10 days recovery. Based on these results we propose a novel concept of inflammatory-induced hibernation of the fetus. Inflammatory priming of fetal metabolism correlated with measures of brain injury, suggesting potential for future biomarker research and the identification of therapeutic targets.

## Introduction

The annual rate of neonatal mortality is approximately four million neonatal deaths worldwide, with the majority resulting from asphyxia (23%), infections (36%), and prematurity (28%) [1]. Preterm birth (delivery before 37 completed weeks of gestation) is the single major risk factor for perinatal mortality and morbidity in both high- and low-income countries. The global annual prematurity rate is 9.6%, representing 12.9 million births [1]. Surviving preterm infants also have high rates of long-term health complications including cerebral palsy, visual or hearing impairment, respiratory illnesses, learning difficulties and behavioral disorders [2].

Prenatal infection or inflammation is a major cause of preterm birth, and can also contribute to poor postnatal growth, pulmonary and neurological morbidity, and mortality [3]–[5]. A causative role for infection *per se* in white matter injury is supported from animal experiments [6]. Further, we recently demonstrated that fetal infection is associated with delayed impairment of both white matter and cortical development [7].

In adult septic patients, sepsis has been shown to be associated with mitochondrial dysfunction in muscle [8]. This condition is considered an important factor regulating multisystem organ failure, morbidity and mortality in septic patients. However, despite the strong support for a key role of prenatal inflammation in neonatal morbidity, the impact of prenatal inflammation on fetal metabolism and bioenergetic failure is unknown. Furthermore, no specific metabolites have previously been shown to correlate with inflammatory-induced developmental brain injury.

Metabolomics can bridge this information gap by elucidating functional information, since metabolite differences in biological fluids and tissues provide the closest link to various phenotypic responses [9]. Metabolomics relies on extensive characterization of the largest possible number of metabolites from relevant or potentially impacted metabolic pathways, and is a promising approach for the clinical investigation of prenatal inflammation. In recent years, electrospray ionization (ESI) tandem mass spectrometry (MS/MS), often applied in concert with an initial chromatography purification/separation step, has been used in a number of clinical metabolomic studies [10], [11]. Generally, metabolomics can be performed in a non-targeted (or “open” profiling) mode using all the information from the spectrometer providing an holistic view of the metabolome with minimal chemical bias, or in so-called “targeted” mode, which relies on analytical protocols optimized to measure specific groups/classes of compounds [12]. Quantitative targeted metabolomics using multiplexed tandem mass spectrometry has matured to the point where it can now be applied in a high-throughput manner [13] and such a targeted approach was applied to this study.

The aim of this study was to examine the effect of inflammation on the plasma metabolome in a model of preterm brain injury in fetal sheep in order to: a) obtain novel descriptive information on dynamic metabolic changes after LPS-induced inflammation, b) investigate the predictive ability of the blood metabolome for brain injury, which could theoretically be useful for development of clinical predictive markers, and c) form the basis for potential individualized therapies in preterm fetuses/babies in the future.

We examined the effect of lipopolysaccharide (LPS) exposure on metabolism in 0.7 gestation fetal sheep, an age equivalent to 28–32 weeks gestation in humans and prior to the onset of cortical myelination [14], [15]. Our data demonstrate that inflammation causes hibernation of fetal metabolism, and that the metabolite response to LPS was strongly associated with cerebral white and grey matter volumes at post mortem. Since metabolic pathways are largely conserved across species and are therefore species-independent, these metabolic fingerprints may translate as potential biomarkers in the clinical setting.

## Results

### Impact of LPS exposure on vital parameters and arterial blood gas analyses

**Figure 1:**
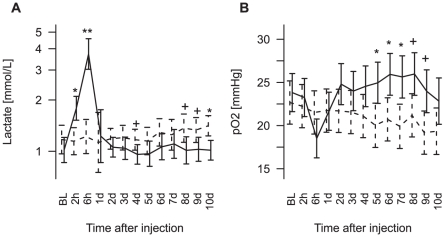
Blood gas parameters. Lactate concentrations (A) and partial pressure of oxygen (B) over the whole course of the experiment. Mean and 95% confidence intervals are given for the LPS (thick line) and the control (dashed line) groups (n = 7 (control)/9 (LPS) +q
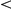
0.2; *, q
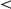
0.05; **, q
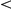
0.001).

The two cohorts had comparable blood gas readings at baseline (p>0.2, Table 1), and no differences in bodyweight, gestational age at LPS injection and post mortem fetal weight (Table 1). Partial pressure of oxygen (pO_2_) and lactate concentration over the course of the experiment are shown in Figure 1. For both parameters, the main time and treatment effects and their interaction were significant. Within the first 6 hours post injection, LPS exposure caused an initial fall in pO_2_ and pH (Figure S1), while lactate was increased 5–fold, compared to the controls. These parameters largely resolved to control group levels by 1 day recovery. In LPS treated animals, lactate was lower from days 8–10 (q<0.05, Figure 1), while pO_2_ was elevated from days 5–9 (q<0.05, Figure 1), when compared to the sham group. Mean arterial blood pressure (MABP) was not significantly different between the groups at any time point [7].

**Table 1:** Description of the cohort.

	Sham (n = 7)	LPS (n = 9)	p-value
pH	7.39 (0.0331)	7.38 (0.029)	0.44
PO2 [mmHg]	22.7 (1.47)	23.8 (3.63)	0.41
sO2	56.2 (6.41)	59.6 (9.52)	0.40
Glucose [mmol/L]	0.771 (0.138)	0.844 (0.142)	0.32
Lactate [mmol/L]	1.2 (0.283)	1.06 (0.324)	0.36
cBase.Ecf [mmol/L]	−1.29 (2.19)	−1.28 (1.82)	0.99
SBC [mmol/L]	22.7 (1.83)	22.8 (1.47)	0.92
Grey matter volume [cm3]	2.79 (0.201)	2.35 (0.284)	0.0037
White matter volume [cm3]	1.47 (0.185)	1.19 (0.121)	0.011
Total brain weight [g]	24.8 (3.65)	22.7 (1.76)	0.20
Fetus weight [g]	1440 (285)	1470 (291)	0.82

Blood gas parameters are measured at baseline and histopathology findings and fetus and brain weights at sacrifice. Statistics given as mean (standard deviation) and p values computed by two sided Welch t-test.

### Acute effects of intrauterine LPS exposure on fetal metabolism

**Figure 2:**
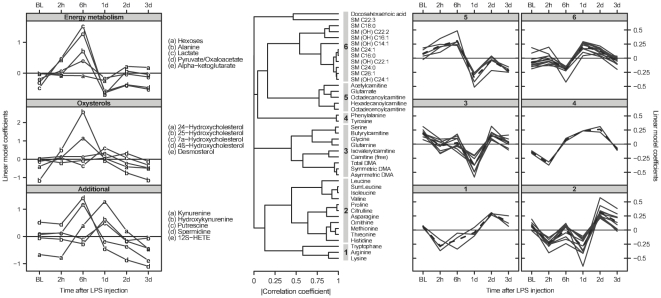
Metabolite response to LPS in the acute phase. Cluster analysis of the time courses for the metabolites altered in the LPS cohort within the first 3 days. Left: display of the time courses for 3 groups of metabolites altered in the LPS cohort within the first 3 days. Middle: dendrogram from the hierachical clustering (HCA), based on the absolute value of the correlation between coefficients of the linear model. Right: explicit display of each metabolite time course for each cluster identified by HCA. The average time course is plotted as a dashed line.

The impact of fetal LPS exposure on the plasma metabolome was first assessed independently in the control cohort (n = 7). However, there were no common regulation patterns, and only very few metabolites exhibited significant trends over the entire experimental course. For example, arachidonic acid, PC aa C36:6, PC ae C36:4, desmosterol and spermidine were the most pronounced down-regulated metabolites over the 10 days recovery (data not shown, FDR<0.01). By contrast, in the LPS group (n = 9), 113 out of 168 metabolites were significantly regulated within the first 3 days (FDR<0.01). Figure S2 shows histograms of the 168 raw p values obtained by time course modelling in the LPS-treated and sham-operated animal cohorts. The patterns of the altered phosphatidylcholines (56 compounds) and the remaining metabolites (57 compounds) are shown in Figure S3 and Figure 2, respectively. Oxysterols, energy metabolism intermediates and other relevant metabolites (Figure 2, left) have been extracted from the rest of the metabolites (Figure 2, right) for ease of interpretation. Clusters comprised a mixture of up- and down-regulated metabolites, with the most extreme variation attaining a more than a 10-fold change in measured plasma concentration. Within the first 3 days, LPS exposure caused a significant increase of the Krebs cycle intermediates, as well as alanine and lactate, and a subsequent slight decrease in hexoses (upper panel, Figure 2, left). Similarly 24-hydroxycholesterol (24OHC) and 25-hydroxycholesterol (25OHC), 12S-HETE and spermidine (lower panel) increased after LPS, peaking at 6 hours. Polyamines, sphingomyelins, amino acids and acylcarnitines also showed specific and distinct patterns after LPS exposure (Figure 2).

### Delayed effects of LPS on plasma metabolome

**Figure 3:**
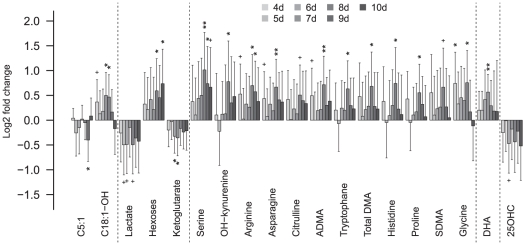
Metabolites altered by LPS after the acute phase. Fold changes (log basis 2) for the metabolites altered at FDR
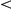
0.1 at any time point after 4 days. Abbreviations: ADMA/SDMA, Asymmetric/symmetric dimethylarginine; Total DMA, sum of ADMA and SDMA; DHA, Docosahexaenoic acid. Significance: + = q
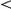
0.2, * = q
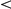
0.05, ** = q
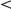
0.001.

We also tested the effects of LPS exposure on plasma metabolites over 3–10 days recovery from LPS. The interaction term denoting differences in concentration between treatment and sampling time identified several metabolites of different classes. Figure 3 (upper left panel) summarizes the number of metabolites that differed between the LPS and control group on at least one time point between days 3–10 in each class at various FDR (solid lines). For example, three energy metabolism intermediates, 10 amino acids, four biogenic amines, two oxysterols, 10 PC's and one prostanoid differed significantly at FDR<0.001. The fold changes of metabolites altered by LPS treatment at day 4 to day 10 is shown in Figure 3 (upper right and lower panel). In LPS exposed animals, 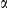-ketoglutarate, lactate and C5:1 were decreased by up to 50%, while hexose and C18:1-OH concentration were increased by up to 80%, compared to the sham animals. The polyamines ADMA, SDMA, the sum of ADMA and SDMA, 3-hydroxykynurenine, kynurenine and spermidine as well as several amino acids were also elevated during the secondary phase in the LPS group compared to the control group, whereas 25OHC was decreased.

### Correlation of plasma metabolites with brain injury

We recently demonstrated in this cohort that fetal LPS exposure causes delayed impairment of both WM and cortical development [7]. Here, we examined correlations of metabolite concentrations with cortical gray (GM) and white matter (WM) volumes at post mortem, as surrogate measures of brain injury. Multivariate analysis using partial least squares regression revealed significant associations (cross validated r = 0.50−0.77, Figure S4) between cortical WM and GM volumes and metabolites levels at 6 h, and 1, 2, 6–9 days, but not at any other time points. We therefore focused on metabolite correlations over an acute phase (6 h–2 d) and a delayed phase (6–9 d). For both periods, we tested the time of sampling and WM and cortical GM volumes as the main effects, as well as their interactions. Density histograms of the raw p values obtained for the correlation analyses of metabolites with (a) GM and (b) WM volumes in the acute (6–48 h) and late phases (6–9 d) are shown in Figure S5.

**Figure 4:**
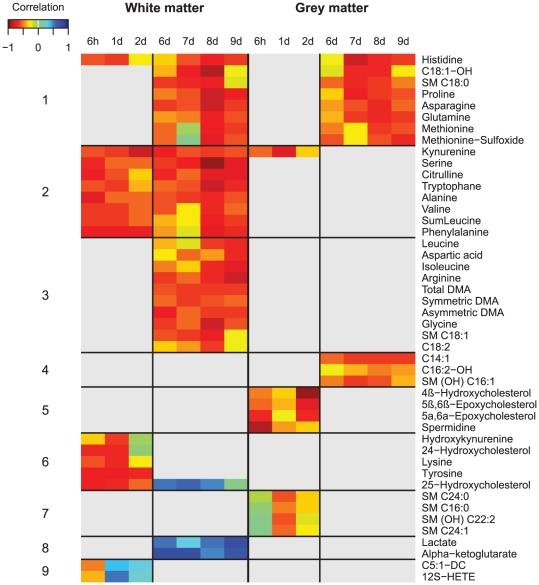
Correlation between metabolites and brain damage. Heatmap depicting metabolite association with white matter and grey matter volumes during the acute phase (6–48 h) and late phase (6–9 days). Color coding follows the key (top right): blue (resp. red) correspond to positive (resp. negative) correlation with the white and gray matter volume (red: increase in metabolite concentration correlates with maldevelopment, blue: decrease in metabolite concentration correlates with maldevelopment, the darker the stronger the correlation). Light grey corresponds to metabolites that were not found to be significant at FDR
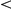
0.1.

Metabolites that exhibited a significant association in at least one sampling window/covariate combination are presented in the form of a heat map of their original correlations to WM/GM (FDR<0.1, Figure 4 and Figure S6 for phosphatidylcholines). In the acute period (6 h–2 d), the associations between metabolites level and cortical GM/WM volumes were largely time dependant. Correlation patterns during this period formed brain region specific clusters (2, 6 and 9 for WM, 5 and 7 for GM). After 6 days, metabolite concentrations correlated with either GM injury (cluster 4) or WM injury (clusters 2,3,6 and 8), or were common to both (cluster 1). Dependent associations could also be detected within the phosphatidylcholines in this period (Figure S6).

## Discussion

Prenatal infection and inflammation are risk factors for preterm birth and increased neonatal morbidity. The role of prenatal inflammation on fetal metabolism and the correlations of fetal metabolism with brain injury are unknown. In this first analysis of a large number of metabolites measured in fetal plasma obtained *in utero*, we established that a single exposure to LPS, a component of gram-negative bacteria, causes long-term metabolic down-regulation and time-specific modulation of endogenous metabolic intermediates correlating with cerebral histopathology in both grey matter and white matter structures.

To ensure that observed changes closely reflect fetal metabolism rather than a maternal response to LPS and/or placenta transfer mechanisms, our model involves direct injection of LPS into a fetal vein and at a much lower dose (some 50 to 500 fold less) than previously reported [16]–[18]. Exposure to this higher and repetitive dosage of LPS resulted in a reduction of placentome in the cross-sectional area and contained areas of injury that could be attributed to placental hypoperfusion and tissue hypoxia. The higher dosing regime was also associated with cardiovascular compromise of the fetus as indicated by acidemia, hypotension and tachycardia [17], [19]. Direct injection of a low dose of LPS into the umbilical fetal vein minimizes the risks of an induced maternal response. However placental effects (and subsequent maternal responses to them) cannot be completely excluded, and neither can any modification of placental metabolite transfer capabilities [20], [21]. Contrary to the transport of respiratory gases that diffuse freely through the placental membranes and whose rate is mainly dependent on concentration gradients and both uterine and umbilical blood flow rates, the transport of nutrients and small molecules is complex [22] and is unlikely to be explained solely by simplistic mechanisms such as passive diffusion [23]. To our knowledge, examples of such processes, particularly for the metabolites discussed here, are scarce. It has been reported that approximately 75% of glucose taken up by the ewe placenta is not transported further to the fetus [24], [25]. Recently, *in vitro* studies demonstrated the placental release of kynurenine into the fetal circulation after LPS exposure, yet at a much higher dose [26]. Altogether, and with the absence of cardiovascular compromise in the fetus [7], the lack of evidence of maternal effects and the fact that arterial blood samples were collected *in utero*, observed metabolite changes are most likely to be indicative of fetal metabolism perturbations [22]. The known functions of some of these metabolites are further discussed here in relation to their potential cause-effect mechanisms.

### Acute LPS effects

LPS exposure was associated with a significant and pronounced change in plasma metabolite levels within hours of administration. The most notable findings are summarized in Table 2. There was an alteration of energy metabolites associated with an acute and transient decrease in arterial oxygen content, with an increase of 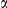-ketoglutarate, an intermediate of the Krebs cycle, alanine, pyruvate, oxaloacetate and lactate, that rapidly resolved to baseline values within 48 hours. Although LPS caused an acute, but transient, decrease in arterial oxygen content, there were no significant changes in heart rate or mean arterial pressure at any time, suggesting that cardiovascular changes were unlikely the major cause of these metabolite changes. This is in accordance with data in septic patients showing an increase in cellular oxygen demand due to LPS treatment, rather than hypoxia due to hypoperfusion [27]. A possible explanation for elevated lactate levels is the increased production of pyruvate as a result of altered pyruvate dehydrogenase (PDH) activity, as described in skeletal muscle tissue during chronic sepsis in rats. Because the inactivation of PDH limits the flux of the substrate through the Krebs cycle, excess pyruvate accumulates in cells and leads to increased production of lactate [28]. Therefore, hyperlactataemia in sepsis is not necessarily evidence of impaired oxygen delivery, but rather reflects the combined effects of pyruvate dehydrogenase inhibition and accelerated glucose transport into cells [28]. Acute changes in mitochondrial respiration (within six hours) have also been demonstrated in laboratory models of sepsis, which were hypothesized to result from nitric oxide and free radical formation due to the inflammatory response [29]. Overall, these data support that inflammation triggers mitochondrial dysregulation in the acute phase of LPS-induced inflammatory response.

**Table 2:** Roles and functions of a selection of altered metabolites.

Acute phase			
Metabolite/parameter	Regulation	Known functions and roles	Relation to LPS exposure
pO_2_	Down	Increased demand in O_2_ in the early stage of sepsis [27]	Increased demand in O_2_
Lactate, 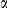 -ketoglutarate pyruvate, oxalo-acetate	Up	Alteration of energy metabolites in sepsis and inflammation, role of mitochondria in the inflammatory response [29], [31]	Increased mitochondrial activity and dysfunction
3-hydroxykynurenine	Down	Production of neuronal damage via the generation ofreactive oxygen species [37]	Increased oxidative stress potentially contributing to brain injury
Kynurenine	Up	Regulator in the innate and adaptive immune response [32]Known excitotoxins in the kynurenine pathway [34]–[36]	Expression of immune response potentially contributing to brain injury
Spermidine, putrescine	Up	Cell growth, proliferation, regeneration and differentiation, regulatory mechanisms of apoptosis [63]–[65]Blood-brain barrier dysfunction in ischemic events [66], [67]HI increases ODC and activates SAT1 [68]–[70]Modification of polyamine levels may be neuroprotective [71]	Increased ornithine decarboxylase (ODC) and SAT1 activities contributing to neuronal injury
25-hydroxycholesterol	Up	Inhibitor of cholesterol biosynthesis, oligodendrocyte cell line morphology and triggered apoptosis [45], [47]	Alterated cholesterol biosynthesis, contributing to oligodendroglial cell death
24-hydroxycholesterol	Up	Crosses blood-brain barrier [39]Marker of brain cholesterol balance, CNS neuronal mass,Alzheimer's disease and multiple sclerosis [40]–[44]Induces inflammatory genes (COX-2, PLA_2_) in neural cells [45], [46]	Altered brain cholesterol biosynthesisInflammatory response
Asymetricdimethylarginine	Down	Endogenous inhibitor of nitric oxide synthase [50]Decreased in the acute phase of sepsis [51]	
Sphingomyelins	Up	Increased sphingomyelinase activity in sepsis [30]	Inflammatory response
**Late phase**			
pO_2_Lactate, 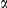 -ketoglutarate,pyruvate, oxalo-acetateHexoses	UpDownUp	Increased demand in O_2_ in sepsis [29]Metabolic down-regulation after inflammation and HI,LPS-induced reduction in mitochondrial biogenesis [52], [53]	LPS-induced hibernation in the fetus as protective adaption to harsh environmental conditions.Potential impact on cortical development
3-hydroxykynurenine	Up	Production of neuronal damage via the generation ofreactive oxygen species [37]	Ongoing inflammatory responsePotential contributor to brain injury
25-hydroxycholesterol	Down	Marker of CNS neuronal mass [40], [41]	Alterated brain cholesterol metabolism
Asymetricdimethylarginine	Up	Increased in chronic vascular disease, augments cardiovascular risk and endothelial dysfunction, biomarker for stroke [48], [49]	Endothelial dysfunction, potentialalteration in blood flow

For each metabolite, known roles and functions are summarized in relation to LPS exposure in the acute and late phases. HI: Hypoxia-ischemia, SAT1: Spermidine/spermine N1-acetyltransferase 1.

These acute alterations in Krebs cycle intermediates were followed by acute phase elevation of sphingomyelins, kynurenine, 3-hydroxykynurenine and putrescine. These metabolites are known to be response markers and mediators of inflammation. The increase in sphingomyelins may reflect sphingomyelinase activity. In fact, sphingomyelinase activity is elevated in septic patients and subjects with organ failure due to activation of the circulating acidic isoform. In animal experiments, increased sphingomyelinase activity is associated with increased apoptosis of liver cells, which can be significantly reduced along with mortality by treatment with sphingomyelinase inhibitors [30]. The pattern of acute phase elevation of inflammatory markers may be explained by the well-established role of mitochondrial reactive oxygen species (ROS) formation in driving pro-inflammatory cytokine production. For example, ROS generated by mitochondrial respiration are important for normal lipopolysaccharide-driven production of several pro-inflammatory cytokines, while pharmacological blockade of mitochondrial ROS efficiently reduces inflammatory cytokine production after LPS stimulation in healthy controls [31].

We also found acute elevation of two members of the kynurenine pathway, kynurenine and 3-hydroxyky-nurenine. This pathway is an important regulator in both innate and adaptive immune responses [32], and increased plasma kynurenine has been suggested to be indicative of immune dysregulation. The kynurenine pathway also has a significant impact on neuronal activity [33]. For example, quinolinic acid can activate a subpopulation of neuronal glutamate receptors specifically sensitive to N-methyl-D-aspartate (NMDA), resulting in neuronal depolarization and excitotoxicity [34]–[36]. 3-hydroxykynurenine, which is redox active, can also cause neuronal damage via the generation of reactive oxygen species [37].

Oxysterols are hydroxylated derivatives of cholesterol that play important functions in lipid metabolism [38], and have been implicated as biomarkers of acute brain damage. In particular, 24-hydroxycholesterol (24OHC) can cross the blood-brain barrier [39], and has been proposed as a biomarker of brain cholesterol balance and as a marker of CNS neuronal mass [40]–[42]. Altered plasma 24OHC has been observed in Alzheimer's disease and Multiple Sclerosis [43], [44]. In addition, 24OHC and 25-hydroxycholesterol (25OHC) play an important role in inflammation and inducing oligodendroglial cell death [45]. 24OHC induces the expression of inflammatory genes such as cyclooxygenase-2 and phospholipase A2 in neural cells [46]. 25OHC, which is synthesized from cholesterol by a specific hydroxylase [47], acts as a potent inhibitor of cholesterol biosynthesis in different cell types. In addition, 25OHC can alter oligodendrocyte morphology and trigger oligodendrocyte apoptosis [45]. Therefore, the increase of 24OHC and 25OHC in the LPS-exposed animals in the acute phase reflects disturbance in cholesterol metabolism and might indicate the onset of neurological injury.

LPS exposure also altered plasma levels of asymmetric dimethyl arginine (ADMA), an endogenous inhibitor of nitric oxide synthase and also associated metabolites of symmetric dimethyl arginine (SDMA). ADMA is elevated in patients with chronic vascular disease, causing increased cardiovascular risk and endothelial dysfunction [48] and are considered potential biomarkers of stroke [49]. The role of ADMA in acute inflammatory states, however, is less well defined, particularly in the fetus and preterm neonate. We found that intrauterine LPS exposure caused a marked and transient decrease in ADMA plasma concentrations during the acute phase, as observed in endotoxaemic rats [50] and in post-operative patients with sepsis without shock [51]. In adult animal models of endotoxaemia, the changes in ADMA over time were suggested to have functional consequences for regulation of microvascular tone [50]. However, in our study there were no changes in mean arterial blood pressure or heart rate during the period of low plasma ADMA levels, and the functional relevance of reduced AMDA in the fetus remains to be elucidated as we did not measure blood flow.

### LPS exposure causes long-term metabolic down-regulation

As well as acute effects, LPS exposure had an impact on long-term fetal metabolism. The key results are summarized in Table 2. During this phase, pO_2_ was significantly increased in the LPS group compared to controls, which was associated with significantly decreased concentrations of lactate (in blood gas analyses and metabolomics), 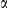-ketoglutarate, and pyruvate/oxaloacetate, and an increase in hexose. These data (hyperglycemia, high pO_2_, low lactate, 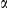-ketoglutarate and pyruvate/oxaloacetate) show that one single LPS exposure alters the bioenergetic status of the fetus towards a hypometabolic condition. This condition has been termed hibernation or metabolic down-regulation in adult septic patients [29]. Hibernation is a suspended animation-like state of reduced metabolic rate and reduced core body temperature. For many animals, this hypometabolic state is a protective adaption to harsh environmental conditions and is a regulated seasonal response. Hibernation has also been described at the cellular level in response to low oxygen tension, ischemic insults and sepsis. In rodent models of sepsis the rate of oxygen consumption by certain tissues was impaired when animals were injected with LPS, and the fall in the rate of oxygen consumption was not due to a change in oxygen delivery (e.g. on the basis of diminished microvascular perfusion), but rather to an acquired intrinsic defect in cellular respiration. A number of different biochemical mechanisms have been postulated including reversible inhibition of cytochrome a,a3 by nitric oxide, irreversible inhibition of one or more mitochondrial respiratory complexes by peroxynitrite [52] and more recently, reduction in mitochondrial biogenesis [53]. Hibernating cells maintain viability by down-regulating oxygen consumption, ceasing cellular function and reducing ATP demand [54]. The metabolic down-regulation in fetuses in our study persisted ten days after LPS exposure, which is more prolonged than the recovery and increased mitochondrial biogenesis reported within 24 hours after LPS exposure in animal models [53]. To the best of our knowledge this is the first time that inflammatory-induced hibernation in the fetus has been described. Future studies are required to examine how long this metabolic down regulation persists.

Our findings have central implications for the treatment of preterm infants previously exposed to intrauterine inflammation. For example, glucose intolerance and postnatal growth retardation are commonly seen in low-gestation newborns, which were often exposed to intrauterine inflammation [3]. Our data might partially elucidate this phenomenon. Secondly, determination of the optimum oxygenation protocols in preterm remains an open question. In cases of metabolic down-regulation the offered oxygen would unlikely be used and might result in an increase in oxidative stress. Current clinical treatment practice and clinical trials assume preterm infants of a given gestational age as one metabolic phenotype/identity. However our data indicate that within a cohort of premature infants, there might be major differences in the metabolic status and availability to use substrates provided, requiring individualized therapy to provide appropriate patient care and treatment.

### Correlation of plasma metabolite levels with brain injury

We previously reported that LPS exposure in this cohort of animals causes maldevelopment of the cortical grey and white matter. In this study, we detected a biphasic pattern of acute and delayed changes in correlating metabolites common to both WM and GM injury, while several groups of metabolites were associated to a specific brain region. Although correlation does not necessarily entail causality, our findings have three implications. First, since the different phases of inflammation could be discriminated, this time-dependent pattern after intrauterine inflammation may enable determination of the current phase of inflammation in an individual preterm infant. These results provide insights into mechanisms and the identification of potential therapeutic targets. Although our analysis was only performed in plasma, the known CNS functions of some of these metabolites indicate cause-effect relationships. For example, novel therapeutic strategies might include inhibition of sphingomyelinases, use of kynurenic acid, an antagonist at several subtypes of glutamate receptors [55]–[57], to counteract the toxicity of quinolinic acid. One limitation of our study in this respect is that we did not measure endogenous levels kynurenic acid, which we previously showed to be neuroprotective in neonatal hypoxic-ischemic brain injury [57]. The modification of oxysterol signaling might be another potential target, since white matter injury in particular, was strongly associated with 24OHC and 25OHC, which can activate pro-apoptotic pathways. Finally, these metabolites might serve as potential biomarkers of developmental brain injury and of treatment effects. Our experiment was not primarily designed for these outcomes, as it would have required tighter control of the false positive discovery rate, a larger animal experiment with pre-defined endpoints, and a verification phase on a human cohort. Nevertheless, we have demonstrated the use of endogenous biochemical intermediates as potential biomarkers, and their relative species-independence suggests appropriate candidates for detecting and monitoring developmental brain injury in a translational scenario.

## Materials and Methods

### Ethics Statement

The experimentation was approved by the Animal Ethical Committee of Gothenburg, Sweden (#307-2006) and conformed to international guidelines on the ethical use of animals. All efforts were made to minimize the number of animals used and their suffering.

### Experimental Protocol

Sheep fetuses were randomly assigned to receive an i.v. bolus infusion of either saline vehicle (Control group; n = 7) or Escherichia coli LPS (055:B4; Sigma-Aldrich, St. Louis, MO; LPS group; n = 9) at 102.5 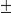 0.5 days of gestation (term = 147 days). The LPS dose was 200 ng/kg Escherichia coli based on a fetal weight of 1 kg at the time of administration. In the first animal models of exposure of the fetus to LPS [18], [19], it was shown that repeated (3–5 times) intravenous injections of high doses of LPS (1 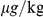) into a fetal vein not only induced hypoxia, acidemia and hypotension of the fetal sheep but also impaired placental function. In our study, the dosage of LPS was lowered to 200 ng/kg in order to mimic subtle chorioamnionitis and to induce brain damage without compromising the fetal cardiovascular system [7]. The fetal sheep were catheterized at 97–99 days of gestation under general anaesthesia (1.5% isoflurane in pO_2_) using sterile techniques. Polyvinyl catheters were placed in both axillary arteries and one axillary vein. The fetal scalp overlying the parasagittal cortex was exposed and two pairs of bilateral holes drilled through the skull (5 and 15 mm anterior of bregma and 0.5 mm lateral of midline). After surgery ewes were housed in individual cages with free access to food and water. Animals were allowed to recover from surgery for at least three days before experimentation, during which time i.v. antibiotics (5 mg/kg gentamycin) were administered to the ewe once daily.

### Tissue and plasma collection

Fetal blood samples were collected for blood gas and metabolomic analyses pre-dose at baseline (BL) and post-dose at 2 hours (2 h), 6 hours (6 h) and every day up to 10 days (1–10 d) after LPS/saline injection. At 10 d post-LPS the ewe and fetus were sacrificed by an i.v. overdose of sodium pentobarbitone. Fetal brains were perfusion-fixed *in situ* with 0.9% NaCl solution then 4% paraformaldehyde in 0.1 M phosphate buffer (Histofix, Histolab, Gothenburg).

### Histology and quantitative analysis of cortical grey and white matter volume

For each animal a series of evenly spaced paraffin embedded coronal sections (10 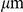, section interval = 150) were collected throughout the forebrain. Between 10–12 brain levels were collected per brain. Sections were stained with acid fuchsin/thionin (AF/T) for quantitative analysis of grey and white matter volume as previously described [7]. In brief, AF/T stained sections were examined for volume changes by light microscopy using StereoInvestigator Software V.7 (Microbrightfield, Inc., Williston, VT). Volumetric changes in cerebral WM (excluding CC) and cerebral cortex were determined using the Cavalieri Principle.

### Metabolomic Analyses

Sample preparation and metabolomic analyses were performed at Biocrates Life Sciences AG, Innsbruck, Austria. Protocols are published elsewhere [58], [59]. In short, multiple reaction monitoring (MRM) transitions specific to 232 endogenous metabolites were monitored using a combination of flow injection and liquid chromatography mass-spectrometry platforms. Quantification was performed using structurally identical or similar labeled compounds that were spiked-in during sample preparation. Samples were randomized within batches and across analytical batches. In addition, several sample aliquots were replicated in all batches to assess batch-to-batch variation. Metabolites that did not meet quality control standards and/or exhibited insufficient abundances were excluded from subsequent interpretation. The final panel comprised 168 unique compounds: 27 acylcarnitines, 15 sphingomyelins, 76 glycerophosphatidylcholines, 22 amino acids, 10 biogenic amines, four prostaglandins, 10 oxysterols and four small organic acids. A detailed list of all metabolites considered in the final analysis is provided in Table S1. All pre-/post-analytical procedures were performed by co-workers blinded to the experimental groups. All methods are validated for human plasma following guidelines documented in the FDA Guidance for Industry-Bioanalytical Method Validation [60].

### Statistical analyses

Data analyses and representations were performed within the statistical environment R. For metabolite measurements, all statistical analyses were performed on pre-processed, log-basis 2 transformed data and reported as such without back-transformation. Estimation of the LPS injection and time effects were computed by generalized least squares regression. Several specifications of the error term were allowed to include the longitudinal structure (i.e. order 1 autocorrelation, Gaussian and exponential spatial correlation structures with time as a continuous covariate) and to deal with potential time-dependent heteroscedasticity. Model selection followed the protocol of Zuur et al. [61]. Fixed effects under consideration were tested with Wald tests with the levels of significance (given as p in the text) adjusted according to Benjamini and Hochberg [62] to control the false discovery rate (FDR). By definition, FDR gives an estimate of proportion of wrongly rejected hypotheses among all significant hypotheses/metabolites selected for interpretation. For each question, distribution of the raw p-values is given in the supplementary materials. Metabolites below the FDR threshold stated in the text are selected for downstream reporting and post hoc testing where p-values are adjusted according to the Holm-Bonferroni method (expressed as q in the text). Unless stated, 95% confidence intervals for the statistic of interest are reported and graphed. Clinical parameters at baseline and the outcome of histology are reported as mean +/− standard deviation, and potential bias in the experimental design was assessed by means of two-sample t tests. Time-dependent changes for altered metabolites are described by the coefficients of the linear model and further grouped by hierarchical clustering using the absolute value of the Pearson correlation between coefficients as the similarity metric. This allows a compact and direct visualization of individual metabolite behavior without losing information about the magnitude of the changes and potential anti-correlation.

## Supporting Information

Figure S1Base deficit and pH over the course of the experiment. Mean and 95% confidence intervals are given for the LPS (thick line) and control (dashed line) groups. Significance summarized as: + = q
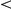
0.2; * = q
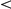
0.05; ** = q
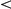
0.001.(TIFF)

Figure S2 Histograms of the 168 raw p values obtained by time course modelling in the LPS-treated and sham-operated animal cohorts. Solid line plotted at y = 8.4 ( = 168*0.05) corresponds to the density to be expected should all metabolites not change over the first 3 days (null hypothesis).
(TIFF)

Figure S3 Time courses of the glycerophospholipids altered in the LPS cohort within the first 3 days post-injection. Left: dendrogram from the hierachical clustering (HCA) based on the absolute value of the correlation between coefficients of the linear model. Right: explicit display of each metabolite time course in the clusters identified by HCA. The average time course is graphed as a dashed line.
(TIFF)

Figure S4 Multivariate correlation between the full metabolite profile (i.e. 168 metabolites) with the white and grey matter volumes analysis by partial least square regression (PLS) at each time point. Y-axis: correlation coefficient (in %) between predicted WM/GM volumes by leave-one-out and actual WM/GM volumes. Negative correlation coefficients are set to 0 for visualisation purposes.
(TIFF)

Figure S5 Density histograms of the raw p values obtained for the correlation analyses on (a) the grey and (b) the white matter volumes at the acute (6–24 h) and late phases (6–9 d). The main effect corresponds to the grey/white matter volume term and interaction to the interaction term between grey/white matter volume and time. The solid line corresponds to the density to be expected should all metabolites are not correlated with the parameter of interest during the given period (null hypothesis).
(TIFF)

Figure S6 Heatmap depicting glycerophosphatidylcholines and their association with white matter (left) and grey matter histology (right) over the two periods 6–48 h and 6–9 days.
(TIFF)

Table S1 Metabolite panel. List of metabolites used in the study.
(PDF)
