# Supplementary figures and images for: Inflammatory-Induced Hibernation in the Fetus: Priming of Fetal Sheep Metabolism Correlates with Developmental Brain Injury
Source: PLoS One. 2011 Dec 29;6(12):e29503. doi: 10.1371/journal.pone.0029503 (PMC3248450; doi:10.1371/journal.pone.0029503)

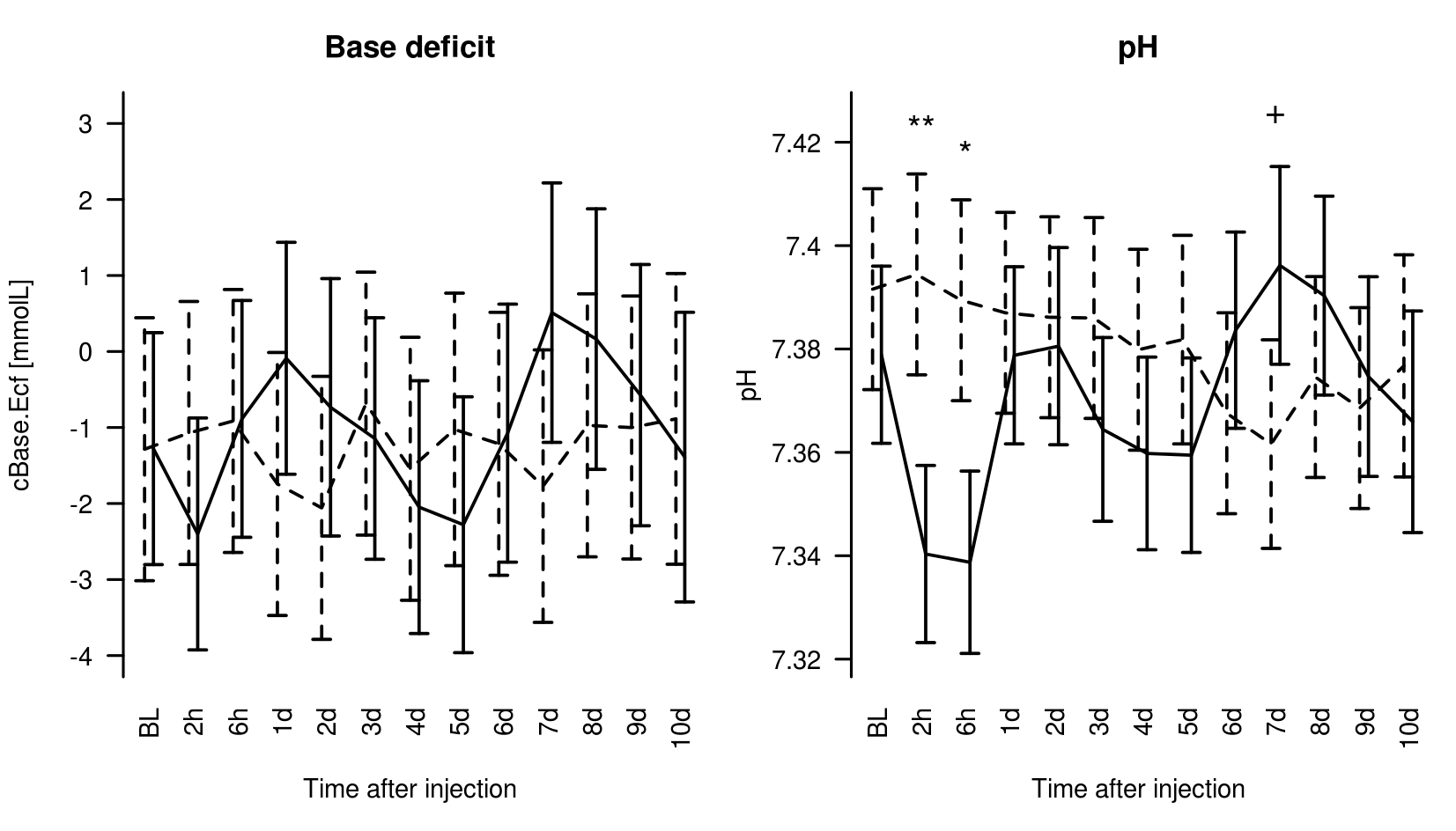

Supplement: Figure S1 — Base deficit and pH over the course of the experiment. Mean and 95% confidence intervals are given for the LPS (thick line) and control (dashed line) groups. Significance summarized as: + = q0.2; * = q0.05; ** = q0.001. (TIFF) [file pone.0029503.s001.tif]

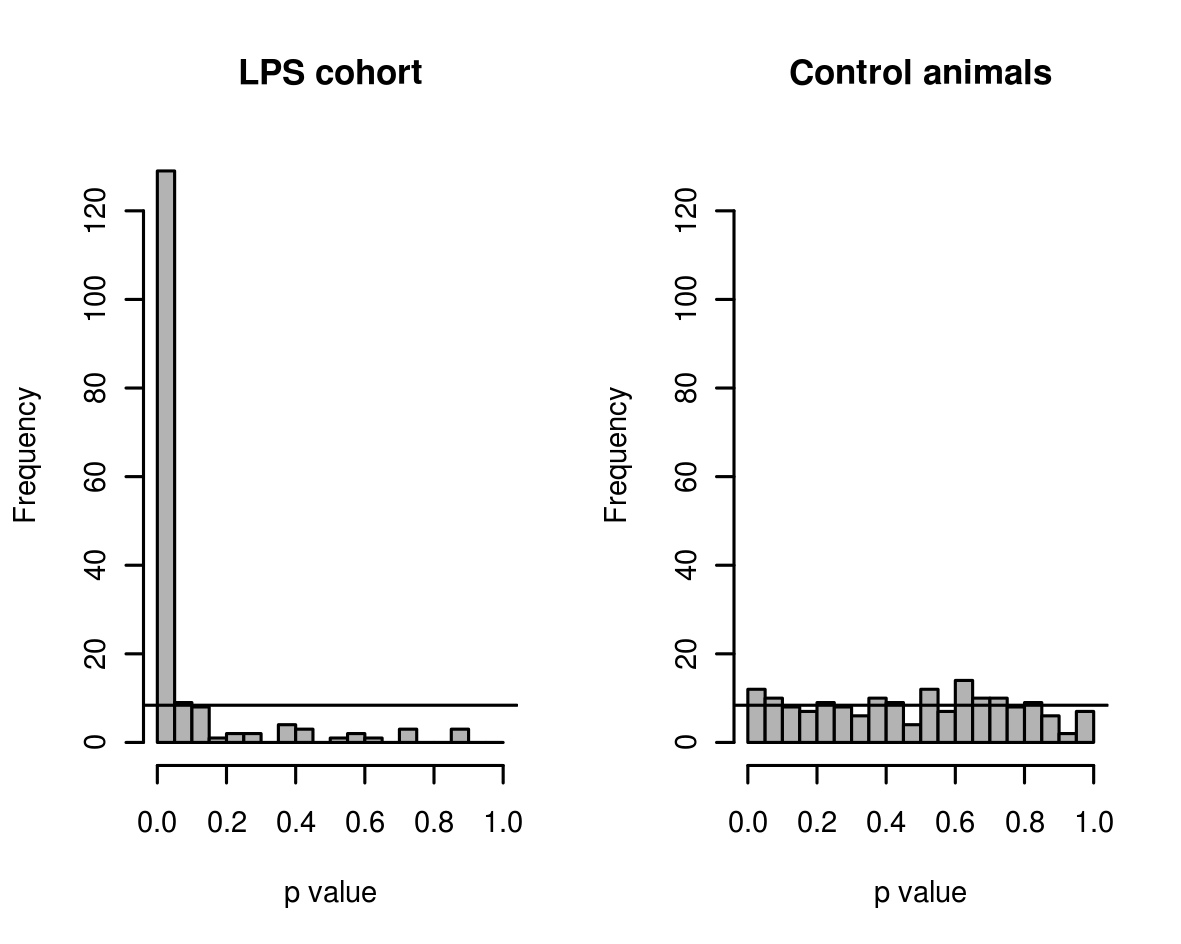

Supplement: Figure S2 — Histograms of the 168 raw p values obtained by time course modelling in the LPS-treated and sham-operated animal cohorts. Solid line plotted at y = 8.4 ( = 168*0.05) corresponds to the density to be expected should all metabolites not change over the first 3 days (null hypothesis). (TIFF) [file pone.0029503.s002.tif]

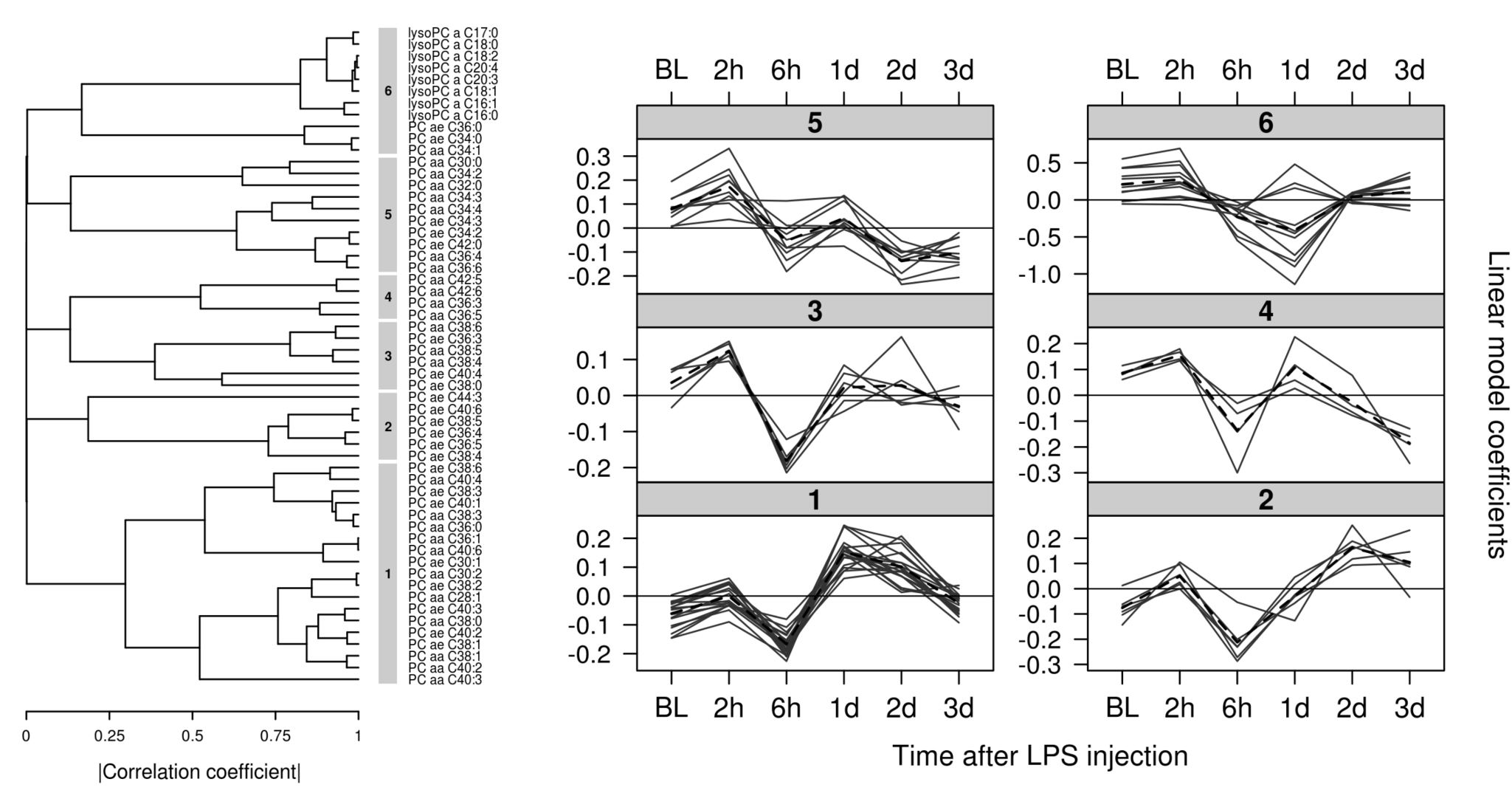

Supplement: Figure S3 — Time courses of the glycerophospholipids altered in the LPS cohort within the first 3 days post-injection. Left: dendrogram from the hierachical clustering (HCA) based on the absolute value of the correlation between coefficients of the linear model. Right: explicit display of each metabolite time course in the clusters identified by HCA. The average time course is graphed as a dashed line. (TIFF) [file pone.0029503.s003.tif]

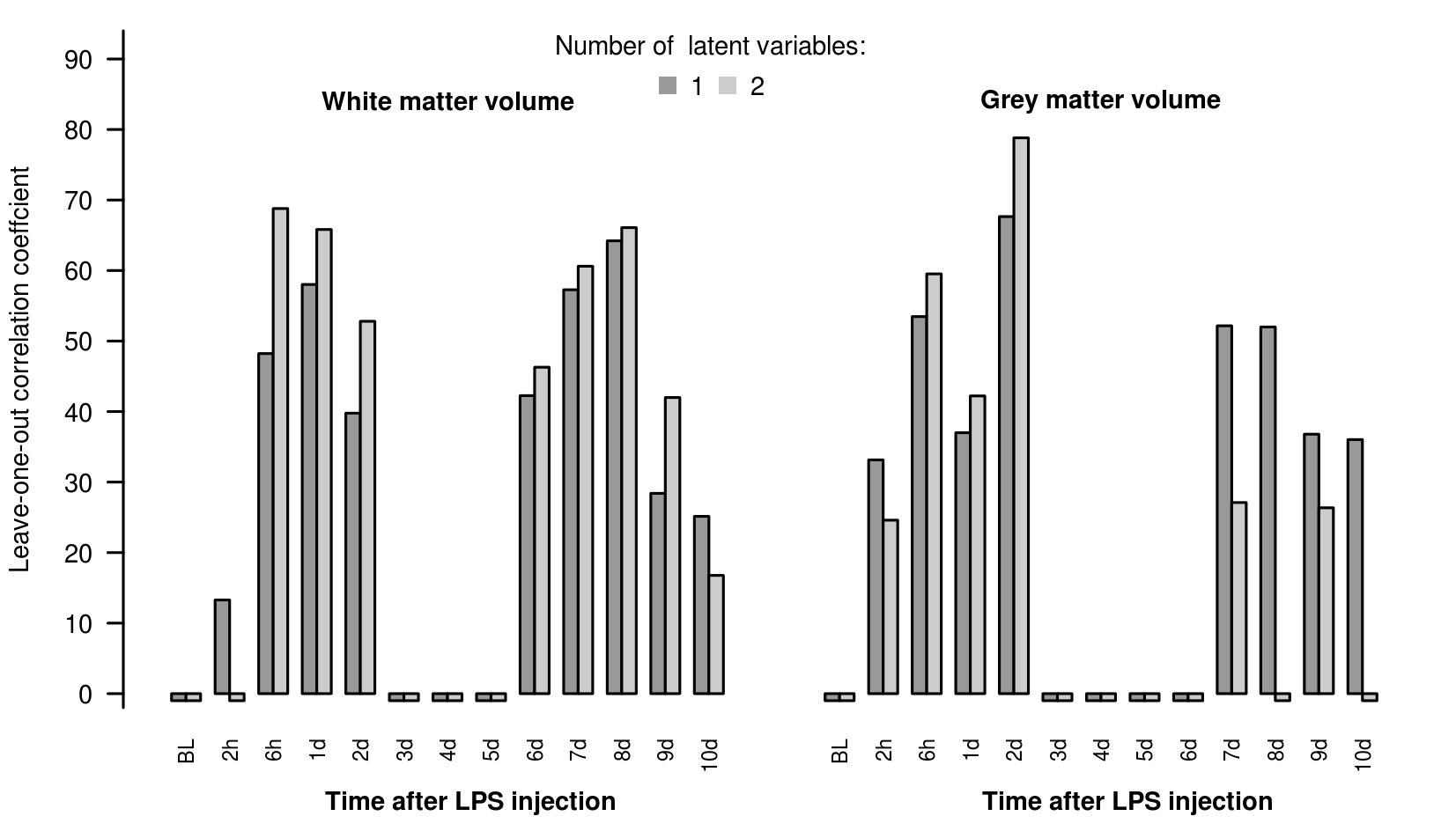

Supplement: Figure S4 — Multivariate correlation between the full metabolite profile (i.e. 168 metabolites) with the white and grey matter volumes analysis by partial least square regression (PLS) at each time point. Y-axis: correlation coefficient (in %) between predicted WM/GM volumes by leave-one-out and actual WM/GM volumes. Negative correlation coefficients are set to 0 for visualisation purposes. (TIFF) [file pone.0029503.s004.tif]

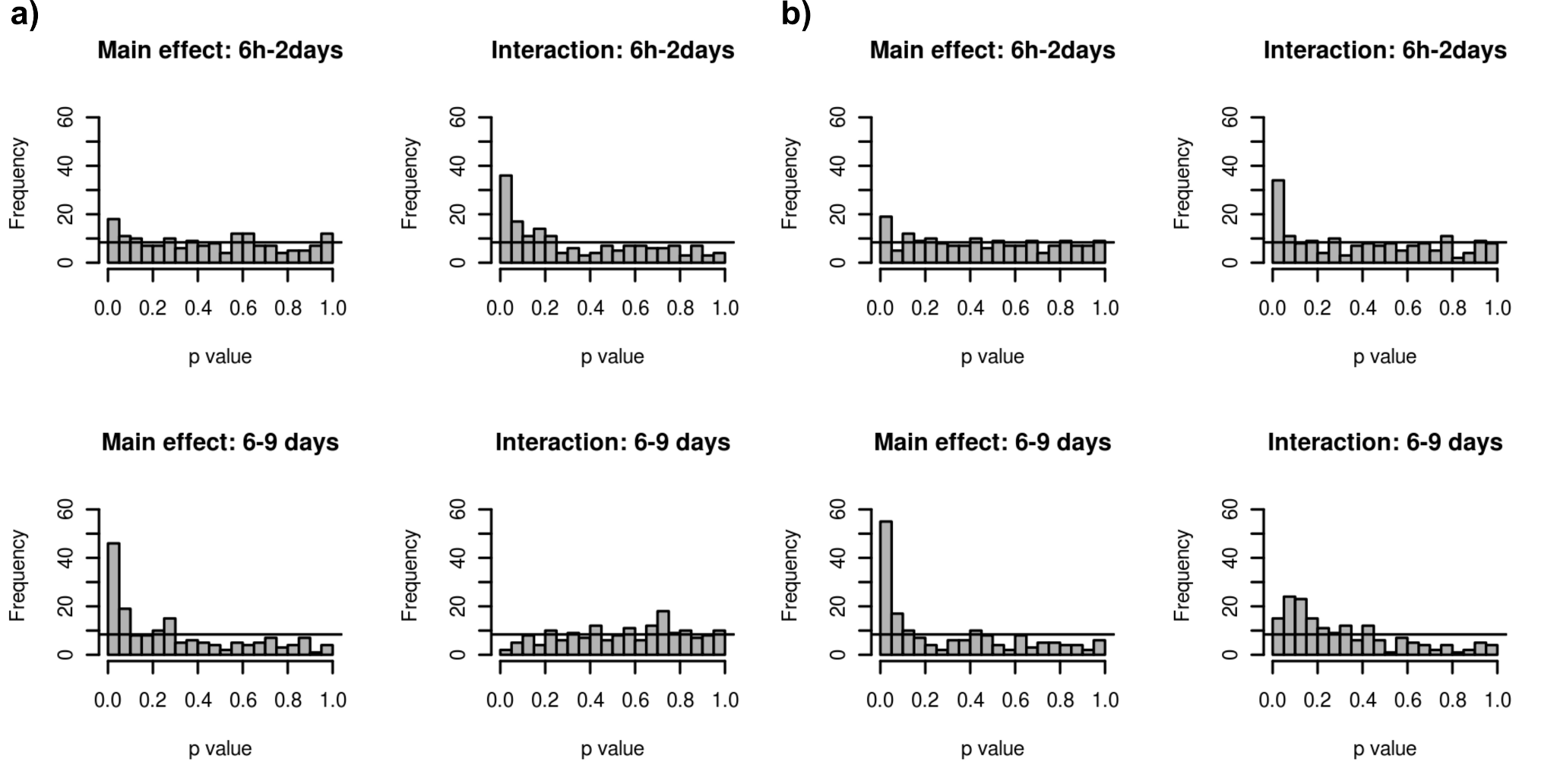

Supplement: Figure S5 — Density histograms of the raw p values obtained for the correlation analyses on (a) the grey and (b) the white matter volumes at the acute (6–24 h) and late phases (6–9 d). The main effect corresponds to the grey/white matter volume term and interaction to the interaction term between grey/white matter volume and time. The solid line corresponds to the density to be expected should all metabolites are not correlated with the parameter of interest during the given period (null hypothesis). (TIFF) [file pone.0029503.s005.tif]

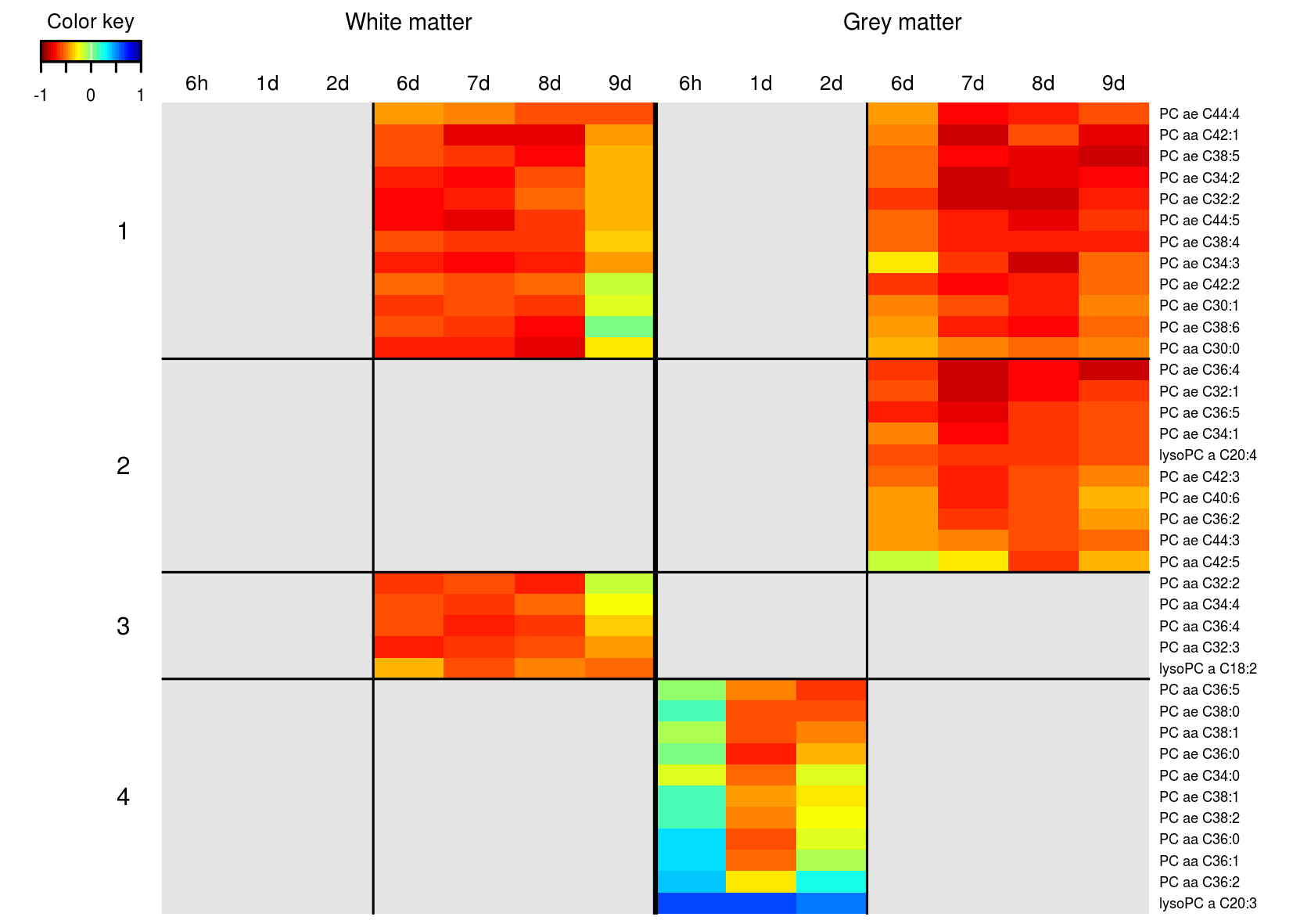

Supplement: Figure S6 — Heatmap depicting glycerophosphatidylcholines and their association with white matter (left) and grey matter histology (right) over the two periods 6–48 h and 6–9 days. (TIFF) [file pone.0029503.s006.tif]
