# Supplementary material for: Inflammatory-Induced Hibernation in the Fetus: Priming of Fetal Sheep Metabolism Correlates with Developmental Brain Injury
Source: PLoS One. 2011 Dec 29;6(12):e29503. doi: 10.1371/journal.pone.0029503 (PMC3248450; doi:10.1371/journal.pone.0029503)
Supplement: Table S1 — Metabolite panel. List of metabolites used in the study. (PDF) [file pone.0029503.s007.pdf]

Amino acids (22)	
Glycine	Glutamine
Alanine	Glutamate
Serine	Methionine
Proline	Histidine
Valine	Phenylalanine
Threonine	Arginine
Isoleucine+Leucine	Citrulline
Leucine	Tyrosine
Isoleucine	Tryptophane
Asparagine	Ornithine
Aspartic acid	Lysine

Energy metabolism (4)	
Hexoses	Pyruvate/Oxaloacetate
Lactate	Alpha-ketoglutarate

Prostaglandines (4)	
13S-HODE	Docosahexaenoic acid
12S-HETE	Arachidonic acid

Biogenic amines (10)	
Asymmetric dimethylarginine	Hydroxykynurenine
Symmetric dimethylarginine	Putrescine
Total dimethylarginine	Spermidine
Methionine-Sulfoxide	Serotonin
Kynurenine	Creatinine

Sphingomyelins (15)	
SM C16:0	SM C26:0
SM C16:1	SM C26:1
SM C18:0	SM (OH) C14:1
SM C18:1	SM (OH) C16:1
SM C20:2	SM (OH) C22:1
SM C22:3	SM (OH) C22:2
SM C24:0	SM (OH) C24:1
SM C24:1	

Oxysterols (10)	
24-S-Hydroxycholesterol	5α,6α-Epoxycholesterol
25-Hydroxycholesterol	4ß-Hydroxycholesterol
27-Hydroxycholesterol	Desmosterol
7α-Hydroxycholesterol	7-Dehydrocholesterol
5ß,6ß-Epoxycholesterol	Lanosterol

Acyl carnitines (27)	
C0 : Carnitine (free)	C12:1 : Dodecenoylcarnitine
C2 : Acetylcarnitine	C14:1 : Myristoleylcarnitine
C3 : Propionylcarnitine	C14:1-OH : 3-Hydroxymyristoleylcarnitine
C3:1 : Propenoylcarnitine	C14:2 : Tetradecadienoylcarnitine
C4 : Butyrylcarnitine / Isobutyrylcarnitine	C16 : Palmitoylcarnitine
C3-DC (C4-OH) : 3-Hydroxybutyrylcarnitine / Malonylcarnitine	C16-OH : 3-Hydroxypalmitoylcarnitine
C4:1 : Butenoylcarnitine	C16:1-OH : 3-Hydroxypalmitoleylcarnitine
C5 : Isovalerylcarnitine / 2-Methylbutyrylcarnitine / Valerylcarnitine	C16:2 : Hexadecadienoylcarnitine
C5-DC (C6-OH) : Glutarylcarnitine	C16:2-OH : 3-Hydroxyhexadecadienoylcarnitine
C5:1 : Tiglylcarnitine / 3-Methyl-crotonylcarnitine	C18 : Stearylcarnitine
C5:1-DC : Glutaconylcarnitine / Mesaconylcarnitine	C18:1 : Oleylcarnitine
C6:1 : Hexenoylcarnitine	C18:1-OH : 3-Hydroxyoleylcarnitine
C7-DC : Pimelylcarnitine	C18:2 : Linoleylcarnitine
C9 : Pelargonylcarnitine	

Glycerophospholipids (76)		
PC aa C24:0	PC aa C40:3	PC ae C38:6
PC aa C28:1	PC aa C40:4	PC ae C40:1
PC aa C30:0	PC aa C40:5	PC ae C40:2
PC aa C30:2	PC aa C40:6	PC ae C40:3
PC aa C32:0	PC aa C42:1	PC ae C40:4
PC aa C32:1	PC aa C42:4	PC ae C40:5
PC aa C32:2	PC aa C42:5	PC ae C40:6
PC aa C32:3	PC aa C42:6	PC ae C42:0
PC aa C34:1	PC ae C30:1	PC ae C42:1
PC aa C34:2	PC ae C32:1	PC ae C42:2
PC aa C34:3	PC ae C32:2	PC ae C42:3
PC aa C34:4	PC ae C34:0	PC ae C42:4
PC aa C36:0	PC ae C34:1	PC ae C44:3
PC aa C36:1	PC ae C34:2	PC ae C44:4
PC aa C36:2	PC ae C34:3	PC ae C44:5
PC aa C36:3	PC ae C36:0	lysoPC a C16:0
PC aa C36:4	PC ae C36:2	lysoPC a C16:1
PC aa C36:5	PC ae C36:3	lysoPC a C17:0
PC aa C36:6	PC ae C36:4	lysoPC a C18:0
PC aa C38:0	PC ae C36:5	lysoPC a C18:1
PC aa C38:1	PC ae C38:0	lysoPC a C18:2
PC aa C38:3	PC ae C38:1	lysoPC a C20:3
PC aa C38:4	PC ae C38:2	lysoPC a C20:4
PC aa C38:5	PC ae C38:3	lysoPC a C6:0
PC aa C38:6	PC ae C38:4	

* Glycero-phospholipids are further differentiated with respect to the presence of ester (a) and ether (e) bonds in the glycerol moiety, where two letters (aa, ea, or ee) denote that the first and the second position of the glycerol scaffold are bound to a fatty acid residue, whereas a single letter (a or e) indicates a bond with only one fatty acid residue; e.g. PC_ae_32:1 denotes a plasmalogen phosphatidylcholine with 32 carbons in the two fatty acid side chains and a single double bond in one of them.
